# New BODIPY Dyes Based on Benzoxazole as Photosensitizers in Radical Polymerization of Acrylate Monomers

**DOI:** 10.3390/ma15020662

**Published:** 2022-01-16

**Authors:** Agnieszka Skotnicka, Janina Kabatc

**Affiliations:** Department of Organic Chemistry, Faculty of Chemical Technology and Engineering, University of Science and Technology, Seminaryjna 3, 85-326 Bydgoszcz, Poland; askot@pbs.edu.pl

**Keywords:** 2-penacylbenzoxazole difluoroboranes, sensitizer, photoinitiator, polymerization, kinetic

## Abstract

A series of 2-phenacylbenzoxazole difluoroboranes named BODIPY dyes (1–8) was designed and applied as photosensitizers (PS) for radical photopolymerization of acrylate monomer. The light absorption within the ultraviolet-visible (UV–Vis) range (λ_max_ = 350–410 nm; ε_max_ = 23,000–42,500 M^−1^cm^−1^), that is strongly influenced by the substituents on the C3 and C4 atoms of phenyl ring, matched the emission of the Omnicure S2000 light within 320–500 nm. The photosensitizer possess fluorescence quantum yield from about 0.005 to 0.99. The 2-phenacylbenzoxazole difluoroboranes, together with borate salt (Bor), iodonium salt (Iod) or pyridinium salt (Pyr) acting as co-initiators, can generate active radicals upon the irradiation with a High Pressure Mercury Lamp which initiates a high-performance UV–Vis light-induced radical polymerization at 320–500 nm. The polymers obtained are characterized by strong photoluminescence. It was found that the type of radical generator (co-initiator) has a significant effect on the kinetic of radical polymerization of acrylate monomer. Moreover, the chemical structure of the BODIPY dyes does not influence the photoinitiating ability of the photoinitiator. The concentration of the photoinitiating system affects the photoinitiating performance. These 2-phenacylbenzoxazole difluoroborane-based photoinitiating systems have promising applications in UV–Vis-light induced polymerization.

## 1. Introduction

In recent years, interest in light-induced polymerization processes has increased rapidly. This advanced technique has resulted in enormous progress in the development of novel adhesives [1,2], photocurable coatings [3], 3D printing [4,5], printing inks [6], preparation of composite materials [7] and many other applications [8,9,10]. Photopolymerization is often considered a “green technology” because it occurs at room temperature, with low energy consumption and with no or almost no release of volatile organic compounds [11]. An effective photoinitiator or photoinitiating system plays a key role in these polymerization processes. That compound absorbs the appropriate wavelengths of light and produces reactive species that induce the rapid conversion of monomers into polymers.

Organoborate salts, diphenyliodonium salts and *N*-alkoxypyridinium salts are commercial compounds which are used as photoinitiators. They absorb light ranging from the from vacuum ultraviolet (UV) to the close UV range, so only UV-light emitting light sources might be used for their excitation. If one wished to apply a longer wavelength light emitting source, addition of a photosensitizer (dye) becomes necessary. In most cases, photoinitiating systems composed of a dye and a co-initiator are highly efficient for radical photopolymerization [12,13,14]. The mechanism of free radical formation is affected by the photoinduced electron transfer process between the photosensitizer and the co-initiator.

Compounds containing BF_2_ moieties represent a class of fluorescent dyes. They are usually characterized by high extinction coefficients, high fluorescence quantum yields, narrow emission bands, relatively long excited-state lifetimes and good photostability [12,15,16]. Among various compounds carrying the BF_2_ moiety the most common are the so-called boron-dipyrromethenes (BODIPYs) [17,18,19,20]. The core of these dyes is a complex of dipyrromethene with a disubstituted boron atom, typically a BF_2_ unit. Tunability of the absorption properties for these type of compounds by changing the substituents at different positions of the BODIPY core is easy.

Phenacylbenzoxazole difluorobonates belong to the class of BODIPY dyes that are widely use in numerous important medical and biochemical applications. These compounds are strongly fluorescent, with blue emissions and very high fluorescence quantum yields and are widely studied in the field of biomedical imaging as immobilized probes [21,22], indicators [23,24], as dyes for biolabeling [25] and chemodosimeters [26]. They are also used in solar cell applications [27]. Furthermore, they can find application in the other fields of technology, for example the polymer industry where the interaction of light with matter is used. Photopolymerization is one of the most important process in that field. It makes use of ultraviolet (UV) or visible (Vis) light to generate reactive species such as radicals or ions [8,28]. They react in a chain reaction. Thus, the photoinitiation of a polymerization process only requires the presence of a molecule capable of absorbing light of an appropriate wavelength (photoinitiator, PI), and the generation of reactive species. Two classes of photoinitiating systems are defined, depending on the mechanism of light conversion into chemical potential (i.e., radicals). In the first class (type I PI), the photoinitiating system contains one molecule (the photoinitiator PI) which is promoted into a dissociative excited state after light absorption and undergoes a homolytic (or heterolytic) cleavage. Type II photoinitiating systems (type II PI) rely on the combination of two molecules. The first molecule absorbs the photon. It is the chromophore and is often called the photoinitiator (PI) or the photosensitizer (PS). The second one could be an electron donor, an electron acceptor or a hydrogen donor [29,30,31,32,33,34,35].

The development of high-performance photoinitiating systems operating under visible light exposure is still a challenge. For this purpose, the use of BODIPY dyes as photosensitizers in light-induced polymerization processes could be a solution.

In 2003 Garcia et al. [5] reported the photopolymerization activity of bimolecular photoinitiator systems that consist of a sensitizer dye, pyrromethene 567 and a radical reagent 3,3′,4,4′-tetra(*ter*t-butylperoxycarbonyl)benzophenone, for the polymerization of 2-hydroxyethyl methacrylate. This type of photoinitiator finds application in the development of new recording materials like holographic memories, photo-imaging and computer to plate devices [5].

Lalevée et al. [36] reported in 2013 the use of photoinitiators based on the BODIPY and boranyl chromophores for the cationic polymerization of epoxy, epoxysilicone and vinyl ether monomers, under irradiation in the 400–600 nm wavelength range. The primary aim was to discover new formulations for the mild synthesis of the corresponding polymers in laminates that would not be affected by atmospheric oxygen. Upon irradiation with visible light these dyes photosensitize the decomposition of diphenyliodonium salts and active species formation. By using a combination of BODIPY and diphenyliodonium hexafluorophosphate, a nearly complete conversion to the desired polyvinyl polymer was observed after irradiation by a laser at 473 nm for a brief time [19,33]. It was also found that the addition of silanes to the photoinitiating system composed of BODIPY and diphenyliodonium salt increases the efficiency of the photopolymerization. In the case of an epoxy monomer, an excellent monomer conversion could be achieved only by making use of a three-component initiating system that incorporated tris(trimethylsilyl)silane as the additional partner. The compositions boranyl/methyldiethanolamine/ phenacylbromide and pyrromethene dye/amine/triazine derivatives were proposed as photoinitiating systems for radical polymerization [34]. However, they initiated trimethylolpropane triacrylate (TMPTA) polymerization with low final conversion [36].

In 2018 Jędrzejewska et al. [37] described photoinitiating systems based on difluoroboranyl dyes of the type NBF_2_O and phenyltriethylborate salt for radical polymerization. It was found that the best photoinitiating system was a mixture: BODIPY dye (1 × 10^−3^ M)/tetramethylammonium phenyltriethylborate salt (7.5 × 10^−3^ M), which under irradiation with light of 100 mW cm^−2^ intensity at 488 nm achieved about 70% conversion of carbon-carbon double bonds after 60 s of irradiation. For other BODIPY/borate salt systems the monomer conversion was in the range from 33.5 to 74% [37].

Lalevée et al. reported in 2019 new boron-pyrromethenes absorbing in NIR light as efficient NIR-photoinitiators in combination with an iodonium salt and phosphine. The BODIPYs have been incorporated into the four-component initiating systems for photopolymerization of methacrylates upon laser diode exposure at 785 nm and 940 nm. The final monomer conversion was higher than 80% after 500 s of irradiation [11].

Recently, Stafford and co-workers [38] described the photoinitiating ability of a series of methine and azo-bridged boron dipyrromethene derivatives acting in the visible to far-red light region [38]. It was found that the halogenation is a simple way that results in about a 5–8-times increase of the polymerization rate at extremally low light intensities (<1 mW cm^−2^), which provides a platform to enable emerging photocuring applications.

**Figure 1:**
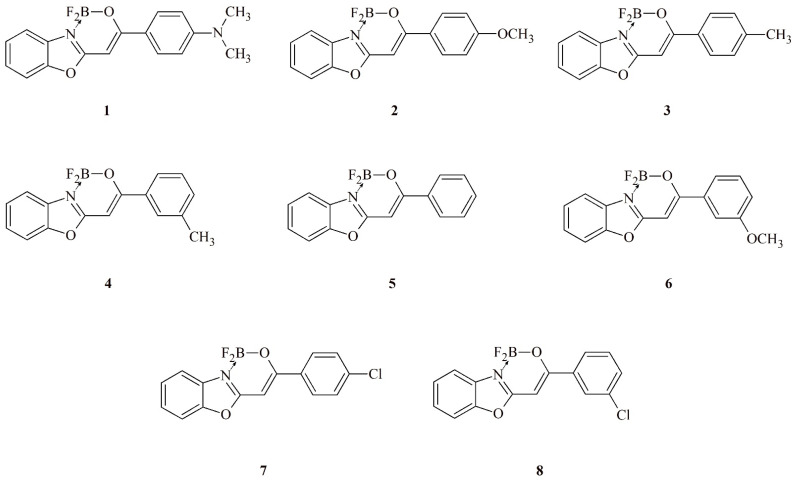
Molecular structure of the prepared 2-phenacylbenzoxazole difluoroboranes.

To our knowledge, benzoxazole-based BODIPY dyes proposed have not yet been applied in photopolymerization reactions, so the present study was undertaken to reveal the polymerization initiation ability of new photoinitiating systems based on difluoroboranyl dyes of the type NBF_2_O and various additives. For this purpose eight 2-phenacylbenzoxazole difluoroboranes with various substituents on C3 and C4 atoms of phenyl ring were designed and synthesized (Figure 1).

The photoinitiation ability and photochemical properties of different photoinitiating systems were studied in detail.

## 2. Materials and Methods

### 2.1. Materials

2-Ethyl-2-(hydroxymethyl)-1,3-propanediol triacrylate (TMPTA), 1-methyl-2-pyrrolidinone (MP), diphenyliodonium hexafluorophosphate (Iod), *N*-methoxy-4-phenylpyridinium tetrafluoroborate (Pyr) and solvents were purchased from Sigma-Aldrich (Poznań, Poland) and used without further purification. Tetramethylammonium *n*-butyltriphenylborate (Bor) was synthesized according to a method described elsewhere [39].

### 2.2. Synthesis

**Scheme 1:**
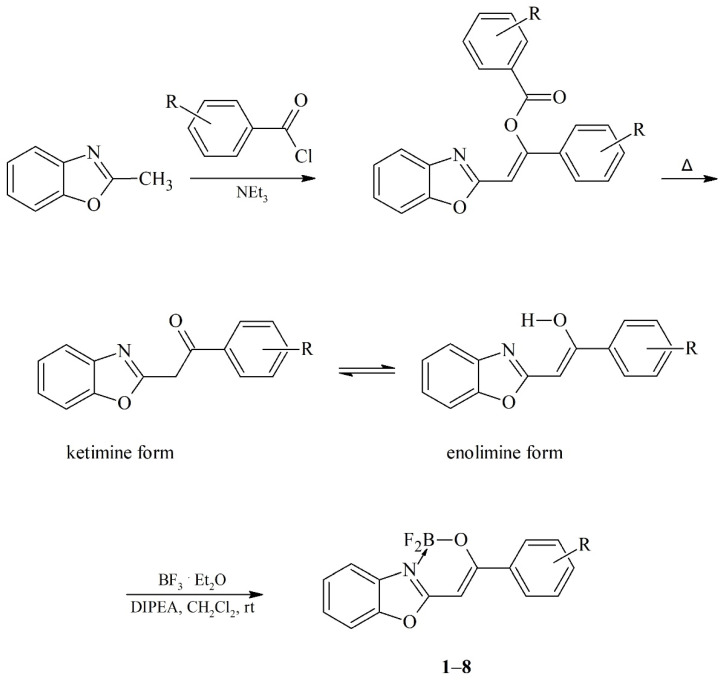
Schematic presentation of the synthesis of 2-phenacylbenzoxazole difluoroboranes (BODIPY dyes where R = 4-NMe_2_ (**1**), 4-OMe (**2**), 4-Me (**3**), 3-Me (**4**), H (**5**), 3-OMe (**6**), 4-Cl (**7**), 3-Cl (**8**)).

The photosensitizers were synthesized by complexationreaction of 2-phenacylbenzoxazole derivative with boron trifluoride etherate (BF_3_) in presence of *N*-ethyldiisopropylamine as is shown in Scheme 1. The details of the synthesis procedures and the correponding ^1^H-, ^11^B-, ^13^C-, ^15^N- and ^19^F nuclear magnetic resonance (NMR) spectra of the products were described previously [40,41].

### 2.3. Spectroscopic Studies

The absorption and emission spectra of synthesized dyes were registered in acetonitrile as a solvent using an UV–Vis Cary 60 spectrophotometer (Agilent Technologies, NS Spectrum, Warsaw, Poland) and a F-7000 spectrofluorometer (Hitachi, Panalityka, Warsaw, Poland), respectively. The measurements were performed at ambient temperature (25 °C) in standard quartz cuvettes (1 cm).

The fluorescence quantum yield of dyes (Φ_fl_) in dichloromethane was determined as follows: first the fluorescence spectrum of diluted (A ≈ 0.1) 2-phenacylbenzoxazole difluoroborane solution was recorded by excitation at the absorption band maximum of the reference. Diluted 9,10-diphenylanthracene in cyclohexane (Φ_ref_ = 0.93) [42] was used as reference. The fluorescence spectrum of 9,10-diphenylanthracene was recorded by excitation at its absorption peak at 355 nm. The fluorescence quantum yield of the tested dyes was estimated based on the following equation (Equation (1)):

(1) $$\Phi_{\mathrm{fl}}=\frac{\Phi_{\mathrm{ref}}A_{\mathrm{ref}}I_{\mathrm{dye}}n_{\mathrm{dye}}^{2}}{A_{\mathrm{dye}}I_{\mathrm{ref}}n_{\mathrm{ref}}^{2}}$$

where Φ_ref_ is the fluorescence quantum yield of the reference sample (9,10-diphenylanthracene) in cyclohexane, A_dye_ and A_ref_ are the absorbance of dye and reference at the excitation wavelength (355 nm), I_dye_ and I_ref_ are the integrated emission intensities for the dye and reference, n_dye_ and n_ref_ are the refractive index of the solvents used for the dye and reference, respectively. Coumarine 153 in cyclohexane (Φ_ref_ = 0.90 [43]; λ_ex_ = 393 nm) was used as reference standard for compound carrying–NMe_2_ group.

### 2.4. Polymerization Measurements

The photopolymerization experiments were carried out by the use of a DSC Q 2000 Differential Scanning Calorimeter (TA Instruments, Warsaw, Poland) a TA Q PCA photo-unit equipped with a high-pressure mercury lamp (Photo–DSC) (TA Instruments, Warsaw, Poland). The measurements were performed under isothermal conditions under a nitrogen flow of 50 mL min^−1^. Radiation in the UV–Vis range (300–500 nm) at a constant intensity equal to 50 mW cm^−2^ was applied as light source.

The polymerization solutions were composed of a sensitizer, appropriate co-initiator, solvent and monomer. The reference sample did not contain a co-initiator. The use of MP was necessary because of the poor solubility of the BODIPY dyes in TMPTA. In general, the samples for polymerization studies were prepared as follows: an appropriate amount of photoinitiator (dye: 1 × 10^−3^ M; co-initiators: 5 × 10^−3^ M and 1 × 10^−2^ M) was dissolved in 0.1 mL of 1-methyl-2-pyrrolidinone, then 0.9 mL of 2-ethyl-2-(hydroxymethyl)-1,3-propanediol triacrylate was added to the solution and mixed. The samples (test sample and reference) of 30 ± 0.1 mg weight were placed into an open aluminum liquid DSC pan and then, maintained at prescribed temperature for 2 min before each measurement run begun. The obtained data were recorded at a sampling interval of 0.05 s per point.

The degree of monomer conversion (C_%_) was calculated on the basis of amount of heat released during polymerization reaction. These liberated heat values are directly proportional to the number of reactive moieties in the monomer molecule (acrylate groups). The conversion percentages were obtained by integrating an area under exothermic peak using Equation (2):

(2) $$C_{\%}=\frac{∆H_{t}}{∆H_{0}^{\mathrm{theor}}}\times 100$$

where: ΔH_t_–the heat evolved at time t during polymerization reaction, $∆H_{0}^{\mathrm{theor}}$—the theoretical heat corresponding to the complete conversion of reactive group. For calculation of C_%_ parameter, the value of theoretical enthalpy for an acrylate double bond polymerization of $∆H_{0}^{\mathrm{theor}}$ = 78.0 kJ mol^−1^ was used.

The rate of polymerization (R_p_) is directly related to the heat flow liberated in reaction and expressed by Equation (3):

(3) $$R_{p}=\frac{\mathrm{dC}}{\mathrm{dt}}=\frac{\mathrm{dH}/\mathrm{dt}}{∆H_{0}^{\mathrm{theor}}}$$

where dH/dt is the heat flow evolved during polymerization reaction.

These kinetic parameters listed above, were calculated for each of investigated systems. Based on the obtained data, the optimal conditions for the polymerization process, in which the photoinitiator works the most effectively, were appointed.

## 3. Results and Discussion

### 3.1. Synthesis and Photophysical Properties

The reaction of 2-methylbenzoxazole with benzoyl chloride and trimethylamine leads to the double benzoylation of the 2-methyl group, that was transformed into –CH = C(Ph)-COOPh. Next, the intermediate product was refluxed in morpholine solution and as a result easily transformed into the corresponding 2-phenacylbenzoxazole derivative. In last step 2-phenacylbenzoxazole reacted with boron trifluoride ethyl etherate in the presence of *N*-ethyldiisopropylamine in dichloromethane and gave the corresponding BF_2_ complexes 1–8 (Scheme 1).

Two series of 2-phenacylbenzoxazole difluoroboranes were designed (Figure 1). The first one included four BODIPY dyes with different substituents on the C4 position of phenyl ring, such as electron-pushing (*N*,*N*-dimethylamino-, methoxy- and methyl-) or electron-withdrawing (chloro-). The second series comprised three dyes with the same groups on the C3 position of the phenyl ring. Both series were compared with the dye possessing a H atom on the phenyl ring.

The crucial parameters in photopolymerization are a compatibility of the PIS absorption spectrum with an emission spectrum of the light source and the number of available incident photons. Thus, the spectral properties of the PI have a big impact on the polymerization rate, Rp, which is directly related with the amount of light absorbed [8,28]. For this reason spectroscopic measurements were made in the beginning.

**Figure 2:**
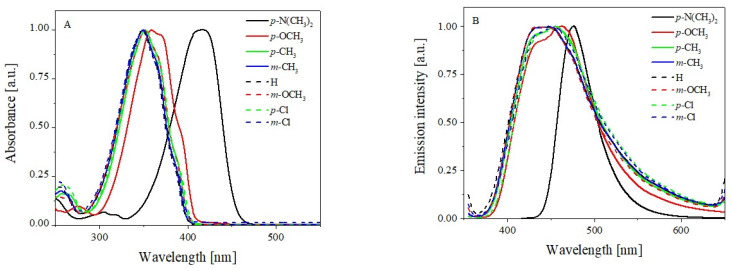
UV-Vis spectra of 2-phenacylbenzoxazole difluoroboranes in acetonitrile as a solvent recorded at room temperature: (**A**): absorption, (**B**): fluorescence at excitation wavelength 355 nm.

Figure 2 presents the absorption and fluorescence spectra of BODIPY dyes when acetonitrile was used as solvent, respectively.

The corresponding spectroscopic data are listed in Table 1.

All 2-phenacylbenzoxazole difluoroboranes exhibited strong absorption and emission in the UV-Vis range, e.g., λ_max_ = 350 nm (except dye **1**), with the molar extinction coefficients (λ_max_) in the range from 23,000 to 42,500 M^−1^cm^−1^, and fluorescence quantum yields (φ_fl_) between 0.005–0.98. For the six 2-phenacylbenzoxazole derivatives (4-NMe_2_, 4-OMe, 4-Me, 3-Me, 3-OMe and H), the maximum absorption peaks (λ_max_) were mainly determined by the phenyl ring substituents, such as 415 nm (4-NMe_2_), 349 nm (H) and 359 nm (4-OMe). The BODIPY dye possessing *N*,*N*-dimethylamino group attached at the *para* position of the phenyl ring shows very interesting spectroscopic properties. A higher absorption 415 nm and emission wavelength 475 nm were found. On the other hand, the lowest Stokes shift of about 3000 cm^−1^, and an outstanding quantum yield 0.98 were obtained. This is a result of the presence of a strong electron-donating group (*N*,*N*-dimethylamino) on the phenyl ring.

As shown in Figure 2 and Table 1, the 2-phenacylbenzoxazole difluoroboranes exhibit absorptions in the visible wavelength range, which allows a good matching with the visible light emission of the Omnicure lamp used.

### 3.2. Photoinduced Radical Polymerization

As shown in Table 1 and Figure 2, the eight 2-fenacylbenzoxazole difluoroboranes showed very good overlap of their absorption spectra with the emission spectrum of the light source used in the photo-DSC experiments. Thus, the free radical benefits of TMPTA upon irradiation with 320–500 nm wavelengths via photo–DSC were monitored in detail.

**Figure 3:**
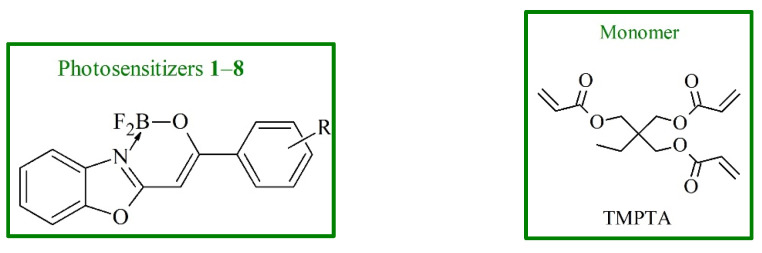

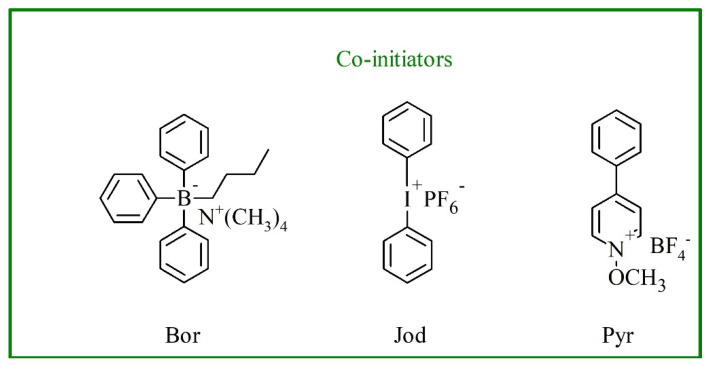
The composition of the polymerizing mixture.

The composition of the polymerizing mixtures composed of photosensitizer, monomer and co-initiators is presented in Figure 3.

#### 3.2.1. Effect of Photosensitizer and Photoinitiating System Structure on the Kinetics of Photopolymerization

**Figure 4:**
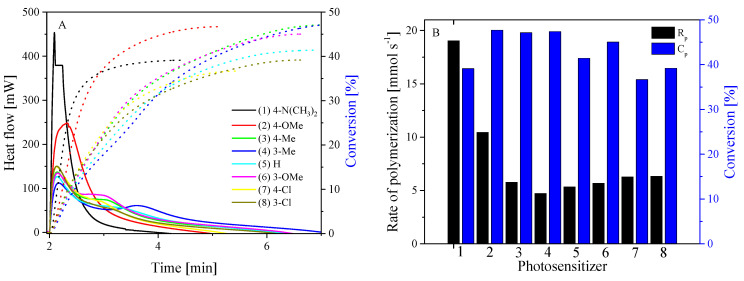
Polymerization profils of TMPTA in the presence of (**A**) 2-fenacylbenzoxazole difluoroboranes/Pyr (1 × 10^−3^ M/1 × 10^−2^ M), (**B**) polymerization of TMPTA in the presence of BODIPY dyes/*N*-methoxy-4-phenylpyridinium tetrafluoroborate under irradiation with UV-Vis light. Light intensity: 50 mW cm^−2^ (solid line denotes data of rate of polymerization; dash line–conversion of monomer).

**Figure 5:**
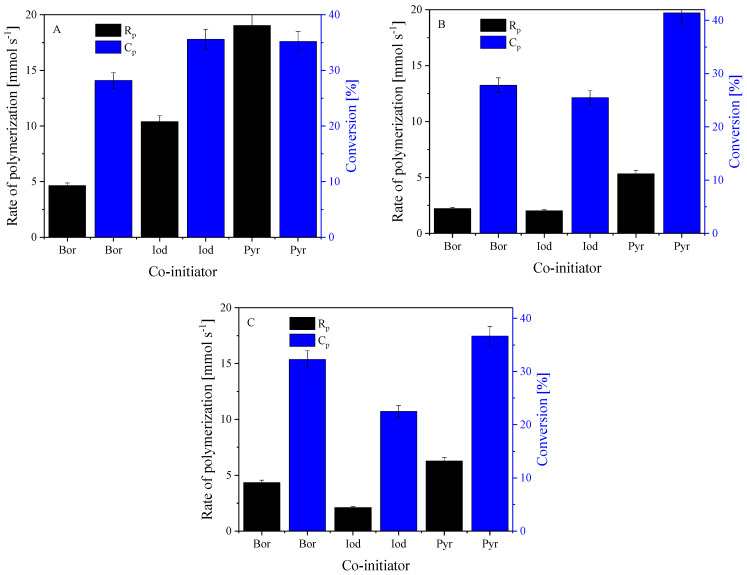
Polymerization of TMPTA in the presence of BODIPY dyes (**A**) (1) 4-N(CH_3_)_2_, (**B**) (5) H, (**C**) (7) 4-Cl, under irradiation with UV-Vis light. Light intensity: 50 mW cm^−2^.

The photoinitiating ability of the systems composed of eight different photosensitizers with three various co-initiators: organoborate salt, iodonium salt and alkoxypyridinium salt were measured and compared. The kinetic results are presented in Figure 4 and Figure 5 and Table 2.

Compared with the 2-phenacylbenzoxazole difluoroborane/co-initiator systems, the BODIPY dyes alone cannot initiate the TMPTA polymerization. All 2-fenacylbenzoxazole difluoroboranes showed good initiation capability together with *N*-alkoxypyridinium salt (Pyr), and the related data are listed in Table 2. The (2)/Pyr photoinitiating system achieved the best monomer conversion and heat flow of 47.7% and 10.45 mmol s^−1^, respectively. For the worst photoinitiating system—**8**/Iod—the monomer conversion and heat flow can reach 21.3% and 1.62 mmol s^−1^, respectively. Compared with other co-initiators studied, diphenyliodonium hexafluorophosphate (Iod)-containing photoinitiating systems preformed the lowest photoinitiating efficiency. The best photoinitiating ability corresponds to the photoinitiating systems composed of 2-phenacylbenzoxazole difluoroborane and *N*-methoxy-4-phenylpyridinium tetrafluoroborate (Pyr). As shown in Figure 4 and Table 2, the monomer conversion of **1**/Pyr, **2**/Pyr, **3**/Pyr, **4**/Pyr, **5**/Pyr, **6**/Pyr, **7**/Pyr and **8**/Pyr were 39.15%, 47.71%, 47.14%, 47.40%, 41.42%, 45.05%, 36.69% and 39.20%, respectively. The heat flows were 19.04 (compound **1**), 10.45 (**2**) and 6.34 (**8**), respectively. The good photoinitiating ability involve tetramethylammonium *n*-butyltriphenylborate (Bor) acting as co-initiator. The best kinetic results were observed for systems: **2**/Bor, **3**/Bor and **4**/Bor. The achieved monomer conversions and heat flows were: 34.38%, 38.24% 38.34% and 4.6 mmol s^−1^, 3.6 mmol s^−1^ and 2.8 mmol s^−1^, respectively.

#### 3.2.2. Effect of Photosensitizer and Photoinitiating System Concentration on the Kinetics of Photopolymerization

**Figure 6:**
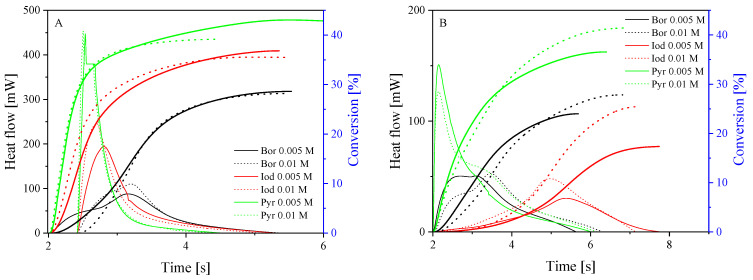
The photopolymerization profils of TMPTA in a presence of (**A**) 1/Bor, 1/Iod, 1/Pyr and (**B**) 5/Bor, 5/Iod and 5/Pyr under irradiation with OmniCure 2000S at different photoinitiating system concentrations (photosensitizer concentration 1 × 10^−3^ M). Light intensity: 50 mW cm^−2^ (solid line denotes data of rate of polymerization; dash line–conversion of monomer).

The following photosensitizers **1** (with the most red-shifted spectra) and **5** (with the most blue-shifted spectra) were chosen here to analyze the effect of the photosensitizer and photoinitiating system concentration. The kinetics of photopolymerization is shown in Figure 6.

The influence of photoinitiating system concentration on photopolymerization performance was analyzed from two points of view. On the one hand, we kept the same concentration of the photosensitizer at 1 × 10^−3^ M, and increased 5-fold the concentration of co-initiator. On the other hand, the concentration of co-initiator was 10 times higher than that of photosensitizer. We found that under 320–500 nm excitation (Figure 6), an increase of the concentration of co-initiator is beneficial to the polymerization. This is because a high concentration of co-initiator produces more free radicals. Therefore, one can conclude that the concentration of photoinitiating system affects the photoinitiating performance.

#### 3.2.3. Effect of the Light Intensity on the Kinetics of Photopolymerization

**Figure 7:**
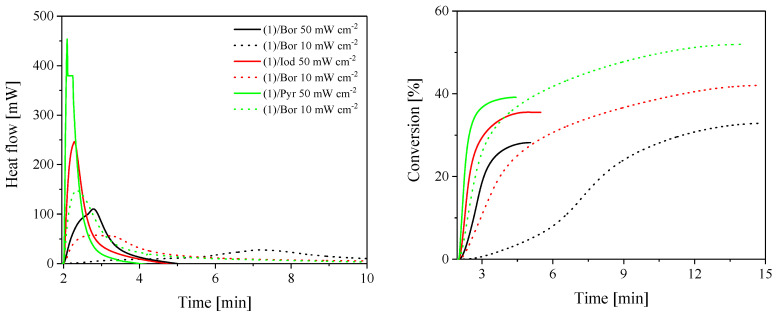
The kinetic curves recorded during radical polymerization of TMPTA monomer initiated by photoinitiating system composed of dye (1) (1 × 10^−3^ M) and co-initiator marked in the figure (1 × 10^−2^ M), light intensity was 10 mW cm^−2^ and 50 mW cm^−2^.

The light source and light intensity influence the kinetics of photoinitiated processes. The effect of the light intensity on the photopolymerization of triacrylate (TMTPA) initiated by systems composed of 2-phenacylbenzoxazole difluoroborane and borate, iodonium and alkoxypyridinium salts was also studied and the recorded kinetic curves are presented in Figure 7.

**Figure 8:**
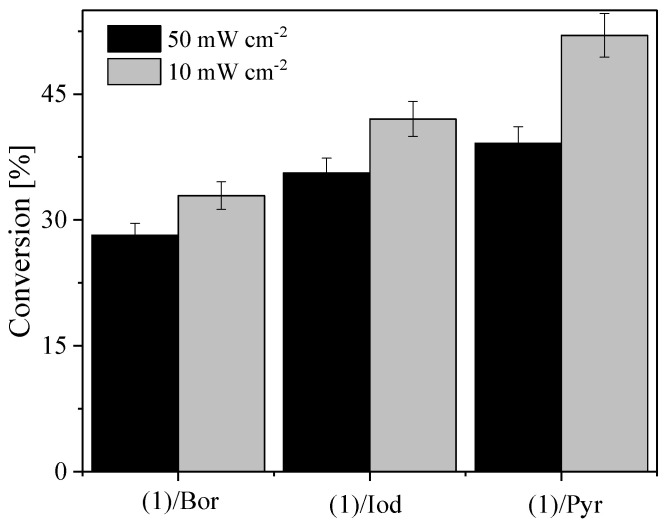
The effect of light intensity on the monomer conversion.

The light intensity influences the kinetics of photopolymerization of acrylate monomer. The higher the light intensity, the higher the polymerization rate were achieved. From Figure 7 it is seen that the highest rates of polymerization were observed for light intensities equal to 50 mW cm^−2^. The decrease of the light intensity to 10 mW cm^−2^ slows down the photopolymerization process and increases the monomer conversion. Figure 8 shows a comparison between the degree of monomer conversion and light intensity (10 mW cm^−2^ and 50 mW cm^−2^). From the economical and environmental point of view this is very important, because of possibility of reduction the consumption of energy needed for polymerization processes.

**Figure 9:**
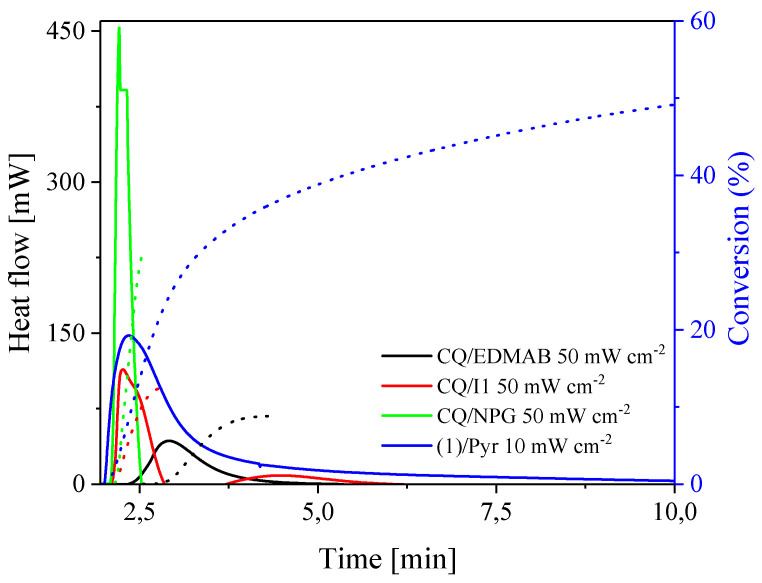
Comparison of TMPTA polymerization initiated by systems: (black) camphorquinone (1 × 10^−3^ M)/ethyl-4-dimethylaminobenzoate (1 × 10^−2^ M), light intensity 50 mW cm^−2^, (red) camphorquinone (1 × 10^−3^ M)/diphenyliodonium chloride (1 × 10^−2^ M), light intensity 50 mW cm^−2^, (green) camphorquinone (1 × 10^−3^ M)/*N*-phenylglycine (1 × 10^−2^ M), light intensity 50 mW cm^−2^, BODIPY dye (1) (1 × 10^−3^ M)/*N*-methoxy-4-phenylpyridinium tetrafluoroborate (5 × 10^−2^ M), light intensity 10 mW cm^−2^ (solid line denotes data of rate of polymerization; dash line - conversion of monomer).

The photoinitiating abilities of the systems composed of 2-phenacylbenzoxazole difluoroboranes and *N*-alkoxypyridinium salt (Pyr) are comparable with the commercial photoinitiators for acrylate photopolymerization, such as camphorquinone (CQ) in the presence of ethyl-4-dimethylaminobenzoate (EDMAB), diphenyliodonium chloride (I1), and *N*-phenylglycine (NPG), as shown in Figure 9.

Based on the kinetic curves of the photopolymerization process (Figure 9) it is seen that the camphorquinone/*N*-phenylglycine system under the same experimental conditions shows the best photoinitiating ability. For this photoinitiator, the monomer conversion value is about 30% under the experimental conditions, which is lower than that achieved with the dye **2**/Pyr photoinitiating system (47.7%).

The lowest efficiency was observed for photoinitiators composed of diphenyliodonium chloride (I1) as a co-initiator. The degree of monomer conversion was low, ranging from 5% to 25% under irradiation with 50 mW cm^−2^ light intensity. Slightly higher conversion values were achieved for the system: BODIPY dye **1** (1 × 10^−3^ M)/*N*-methoxy-4-phenylpyridinium tetrafluoroborate (5 × 10^−2^ M) when the light intensity was 10 mW cm^−2^.

**Figure 10:**
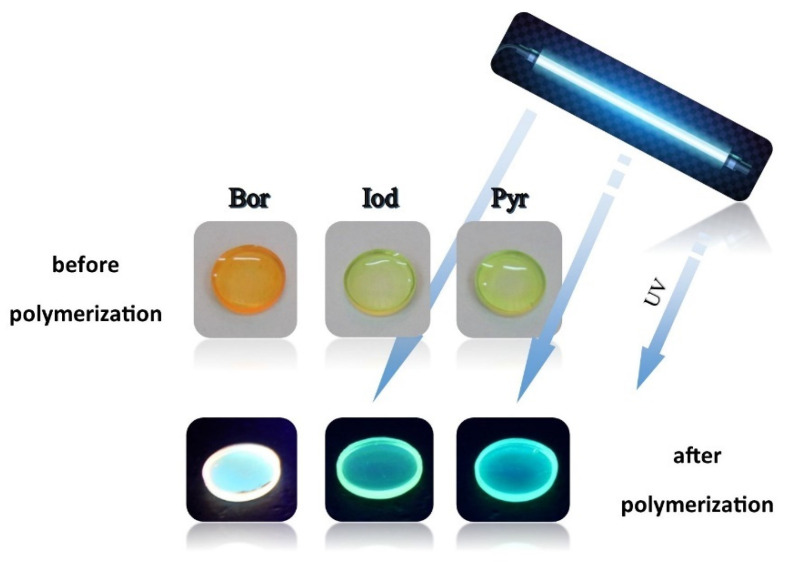
Images of the samples before and after photopolymerization process.

To sum up, the new photoinitiating systems are efficient and yielded a good final conversion of TMPTA, indicating the systems’ potential applications in the creation of coatings. Figure 10 shows images for the samples before and after the photopolymerization process.

Notably, the final products (polymers) obtained after photopolymerization processes initiated by BODIPY dyes and borate salt, diphenyliodonium salt or *N*-alkoxypyridinium salts are photoluminescent. Therefore, the photoinitiating systems composed of 2-phenylbenzoxazole difluoroboranes may be applied for the preparation of strongly photoluminiscent coatings and layers.

## 4. Conclusions

In summary, eight 2-phenacylbenzoxazole difluoroboranes were designed and synthesized by introducing different substituents into the C3 or C4 position of the phenyl ring. All BODIPYs displayed strong absorption within the near UV-Vis light range due to ππ*-transitions. The eight new 2-phenacylbenzoxazole difluoroboranes efficiently initiate the free radical polymerization of 2-ethyl-2-(hydroxymethyl)-1,3-propanediol triacrylate (TMPTA) when irradiated with Omnicure S2000 lamp within the 320–500 nm range in the presence of borate, iodonium or alkoxypyridinium salts. *N*-Alkoxypyridinium salt performed better than the other co-initiators studied. Compared with commercial photoinitiating systems containing camphorquinone as a photosensitizer, the selected 2-phenacylbenzoxazole difluoroboranes performed better in the 320–500 nm wavelength irradiation range. The crucial effects on polymerization rate are the concentration of both components of the photoinitiating system and the light intensity. The 2-phenacylbenzoxazole derivative-based photosensitizers can be used in photopolymerization of acrylate monomers under UV to visible light.

## Figures and Tables

**Table 1:** Spectroscopic properties of photosensitizers in acetonitrile as solvent.

Dye	Substituent	λ_abs_ [nm]	λ_fl_ [nm]	Stokes Shift [cm^−1^]	Φ_fl_ [× 10^−2^] *
**1**	4-NMe_2_	415	475	3040	98.2
**2**	4-OMe	359	463	6260	1.8
**3**	4-Me	351	455	6500	0.66
**4**	3-Me	350	448	6300	0.48
**5**	H	349	447	6300	0.54
**6**	3-OMe	351	434	5400	0.57
**7**	4-Cl	352	455	6400	0.58
**8**	3-Cl	350	452	6400	0.50

* Measured in dichloromethane.

**Table 2:** The Kinetic Data of TMPTA Photopolymerization.

Dyes	Bor		Iod		Pyr	
	R_p_ [mmol s^−1^]	C_%_ [%]	R_p_ [mmol s^−1^]	C_%_ [%]	R_p_ [mmol s^−1^]	C_%_ [%]
**1**	4.65	28.2	10.37	35.6	19.04	39.2
**2**	4.60	34.4	2.12	25.9	10.45	47.7
**3**	3.60	38.2	1.75	23.3	5.78	47.1
**4**	2.80	38.3	1.61	24.5	4.73	47.4
**5**	2.22	27.8	2.02	25.5	5.34	41.4
**6**	3.42	35.2	1.81	22.6	5.68	45.1
**7**	4.35	32.3	2.11	22.5	6.28	36.7
**8**	4.01	37.1	1.62	21.3	6.34	39.2

* The concentration of photosensitizer and co-initiator was 1 × 10^−3^ M and 5 × 10^−2^ M, respectively.

## Data Availability

Data sharing is not applicable for this article.
